# Predictive value of tyrosine phosphatase receptor gamma for the response to treatment tyrosine kinase inhibitors in chronic myeloid leukemia patients

**DOI:** 10.1038/s41598-021-86875-y

**Published:** 2021-04-23

**Authors:** Mohamed A. Ismail, Marzia Vezzalini, Hisham Morsi, Ahmad Abujaber, Ali Al Sayab, Kodappully Siveen, Mohamed A. Yassin, Maria Monne, Muthanna Samara, Richard Cook, Claudio Sorio, Helmout Modjtahedi, Nader I. Al-Dewik

**Affiliations:** 1 School of Life Science, Pharmacy and Chemistry, Faculty of Science, Engineering and Computing, Kingston University London, London, United Kingdom; 2 Interim Translational Research Institute (iTRI), Hamad Medical Corporation (HMC), Doha, Qatar; 3 Department of Medicine, University of Verona, Verona, Italy; 4 Quality of Life Unit, National Center for Cancer Care and Research, (NCCCR), Hamad Medical Corporation (HMC), Doha, Qatar; 5 Department of Medical Oncology, National Centre for Cancer Care and Research, Hamad Medical Corporation (HMC), Doha, Qatar; 6 Centro di Diagnostica Biomolecolare e Citogenetica Emato-Oncologica, “San Francesco” Hospital, Nuoro, Italy; 7 Department of Psychology, Kingston University London, Penrhyn Road, Kingston upon Thames, KT1 2EE United Kingdom; 8 Qatar Medical Genetic Center (QMGC), Hamad General Hospital (HGH), and Interim Translational Research Institute (iTRI), Hamad Medical Corporation (HMC), P.O. BOX. 3050, Doha, Qatar; 9 College of Health and Life Science (CHLS), Genomics and Precision Medicine, Hamad Bin Khalifa University (HBKU), Doha, Qatar

**Keywords:** Oncology, Pathogenesis, Oncogenes, Cancer, Cancer therapy, Cancer therapeutic resistance, Targeted therapies

## Abstract

Protein tyrosine phosphatase receptor gamma (*PTPRG*) is a member of the receptor-like family protein tyrosine phosphatases and acts as a tumor suppressor gene in different neoplasms. Recent studies reported the down-regulation of *PTPRG* expression levels in Chronic Myeloid Leukemia disease (CML). In addition, the *BCR-ABL1* transcript level is currently a key predictive biomarker of CML response to treatment with Tyrosine Kinase Inhibitors (TKIs). The aim of this study was to employ flow cytometry to monitor the changes in the expression level of PTPRG in the white blood cells (WBCs) of CML patients at the time of diagnosis and following treatment with TKIs. WBCs from peripheral blood of 21 CML patients were extracted at diagnosis and during follow up along with seven healthy individuals. The PTPRG expression level was determined at protein and mRNA levels by both flow cytometry with monoclonal antibody (TPγ B9-2) and RT-qPCR, and *BCR-ABL1* transcript by RT-qPCR, respectively. PTPRG expression was found to be lower in the neutrophils and monocytes of CML patients at time of diagnosis compared to healthy individuals. Treatment with TKIs nilotinib and Imatinib Mesylate restored the expression of PTPRG in the WBCs of CML patients to levels observed in healthy controls. Moreover, restoration levels were greatest in optimal responders and occurred earlier with nilotinib compared to imatinib. Our results support the measurement of PTPRG expression level in the WBCs of CML patients by flow cytometry as a monitoring tool for the response to treatment with TKIs in CML patients.

## Introduction

Chronic myeloid leukemia (CML) accounts for 15–20% of all hematological malignancies amongst adults^1^. BCR/ABL is the first oncoprotein discovered in patients with CML and the first target for therapeutic intervention with small molecule tyrosine kinase inhibitors (TKIs) based drugs^2^. To date, five different BCR-ABL TKIs (i.e., Imatinib Mesylate (IM), Nilotinib, Dasatinib, Ponatinib, and Bosutinib) have been approved by the United States Food and Drug Administration (FDA) for the treatment of patients with CML^3^. However, primary or secondary resistance to treatment with TKIs occur in some patients with CML, highlighting the need for better understanding the mechanisms of resistance and identification of the predictive biomarkers for the response to treatment^4^.

Protein Tyrosine Phosphatase Gamma (PTPRG) is a tumor suppressor and a member of a family of receptors tyrosine phosphatases^5^. It has the ability to remove a phosphate group from the phosphorylated amino acid tyrosine that is present on its substrate protein, thus balancing the ABL1 tyrosine kinase activities^6^. PTPRG is located on the short arm (3p14.2) of chromosome 3 and consists of 30 exons. It has an extracellular domain, a transmembrane domain, a carbonic anhydrase-homologous amino terminus followed by a fibronectin III domain, and two intracellular PTPase catalytic domains of which the only one is active^7^. *PTPRG* regulates hematopoietic differentiation processes and is expressed in human hematopoietic precursor cells, as well as in neutrophils and monocytes^8^.

In 2010, Della Peruta et al. reported down-regulation PTPRG at both mRNA and protein levels in leukocytes of CML patients^9^. The status of PTPRG has also been reported to be important in the response to treatment with TKIs in CML patients^10^. In another study, our group identified a single Nucleotide Polymorphism (SNPs) (rs62620047) in *PTPRG* (Y92H) in patients who failed Imatinib Mesylate (IM) treatment^11^. More recently, hypermethylation of *PTPRG* loci was reported to be a molecularly independent mechanism of resistance to treatment with TKI in CML patients^12^.

Flow cytometry is a simple and robust technique employed in many laboratories for the diagnostic and research purposes. It can also be of potential value in the monitoring of CML patients and their response to therapeutic intervention^13–16^. In a previous study, we described the production of a monoclonal antibody that recognizes the extracellular domain of PTPRG (TPγ B9-2) on CML cell lines and patients’ samples. This is a unique antibody with a potential capacity for monitoring the PTPRG expression in CML patients^17^.

In this study, using flow cytometry, we determined the expression levels of PTPRG amongst a sub-population of white blood cells in healthy individuals and CML patients at the time of diagnosis and following treatment with TKIs. We also compared the effect of TKIs on *BCR-ABL1* and *PTPRG* transcripts via Reverse Transcriptase PCR (RT-qPCR).

## Results

To our knowledge, mAb TPγ B9-2 is currently the only mAb for use in the detection of PTPRG protein by flow cytometry. Therefore, in this study, the changes in the expression level of PTPRG protein were determined in the WBCs of seven healthy individuals and 21 CML patients at the time of diagnosis and following treatment with BCR-ABL TKIs, using flow cytometry.

### Characteristics of CML patients at the time of diagnosis, their responses to the treatment with TKIs and healthy control participants

Out of the 21 CML patients examined in this study, 18 (86%) were diagnosed at chronic phase (CP) and 3 (14%) at accelerated phase (AP)^18^. The mean age of the 21 CML patients was 38.21 years and those of seven healthy controls 35.2 years. Eleven patients were treated with Imatinib (400 mg) and of these only two patients had optimal responses. Optimal response has also been developed in one patient following treatment with 600 mg of Imatinib. Out of the remaining nine CML patients treated with Nilotinib (300 mg), seven patients had optimal responses and the remaining two patients failed responses (Table 1). Overall, out of 21 CML treated patients with TKIs, 11 patients had optimal responses (52%), and ten patients had failed treatment (48%), (Table 1). In addition, the results of Fisher's Exact Test revealed that treatment with Nilotinib (300 mg) was more likely to lead to optimal response compared to treatment with Imatinib (400 mg) (Odds Ratio: 15.75, 95% CI 1.75–141.41, Z: 2.46, p < 0.05) (Table 2A).

**Table 1:** CML patient's characteristics: gender, age, clinical phase, TKIs (BCR/ABL1 and PTPRG) at diagnosis stage and response to treatment.

Patients	Gender	Age (years)		Diagnosis		BCR-ABL1(IS) (%)	PTPRG (%)	Treatment	Final response
CML 01	M	61		CP		37	0.02	Imatinib (400 mg)	Failed
CML 02	M	61		CP		100	0.03	Imatinib (400 mg)	Optimal
CML 03	F	33		CP		100	0.02	Nilotinib (300 mg)	Optimal
CML 04	M	46		AP^a^		100	0.01	Nilotinib (300 mg)	Optimal
CML 05	M	23		CP		100	0.01	Imatinib (400 mg)	Failed
CML 06	M	48		CP		100	0.02	Imatinib (400 mg)	Optimal
CML 07	M	43		CP		100	0.02	Imatinib (400 mg)	Failed
CML 08	M	36		CP		100	0.01	Imatinib (400 mg)	Failed
CML 09	M	48		CP		100	0.02	Imatinib (400 mg)	Failed
CML 10	M	45		CP		100	0.02	Imatinib (400 mg)	Failed
CML 11	M	26		CP		100	0.01	Imatinib (400 mg)	Failed
CML 12	F	28		CP		100	0.01	Nilotinib (300 mg)	Optimal
CML 13	M	40		CP^b^		100	0.01	Imatinib (600 mg)	Optimal
CML 14^d^	M	45		CP		100	0.01	Nilotinib (300 mg)	Failed
CML 15	M	26		CP		100	0.01	Imatinib (400 mg)	Failed
CML 16	M	40		CP		100	0.01	Imatinib (400 mg)	Failed
CML 17	M	33		CP		89	0.02	Nilotinib (300 mg)	Optimal
CML 18	M	34		CP		100	0.02	Nilotinib (300 mg)	Optimal
CML 19	M	32		AP		100	0.01	Nilotinib (300 mg)	Optimal
CML 20^d^	M	65		AP^c^		100	0.01	Nilotinib (300 mg)	Failed
CML 21	M	40		CP		100	0.01	Nilotinib (300 mg)	Optimal

^a^CML04 AP with additional chromosomal t(9:22) (q34, q11.2); t(11; 14) (q23, q32) (30).

^b^CML13 CP with Tuberculosis.

^c^CML20 AP with double Ph + .

^d^CML14 patient lost to record myeloid lineage events at time of relapsed.

**Table 2:** (A) Contingency table of TKIs therapy with outcome response. *CML patients with IM 600 mg excluded from fisher exact estimation. (B) Test Statistics of effect of Nilotinib and Imatinib Mesylate on neutrophils.

(A) TKI therapy	Optimal response	Failure response	Total	
Imatinib Mesylate (400 mg)	2	9	11	
Nilotinib (300 mg)	7	2	9	
Total	9	11	20	

(B) Time points	Nilotinib (300 mg)		Imatinib Mesylate (400 mg)	
	Difference in mean rank	*P* value	Difference in mean rank	*P* value
Diagnosis-1st follow up	− 1.25	*p* = 0.03	− 0.83	*p* = 0.69
Diagnosis-2nd follow up	− 2.38	*p *˂ 0.001	− 2.083	*p *˂ 0.001
Diagnosis-3rd follow up	− 2.38	*p *˂ 0.001	− 2.75	*p *˂ 0.001
1st follow up–2nd follow up	− 1.13	*p* = 0.49	− 1.25	*p* = 1.06
1st follow up–3rd follow up	− 1.13	*p* = 0.488	− 1.91	*p *˂ 0.002
2nd follow up–3rd follow up	0.00	*p* = 1.0	− 0.667	*p* = 1.0

### Presentation of results

**Figure 1:**
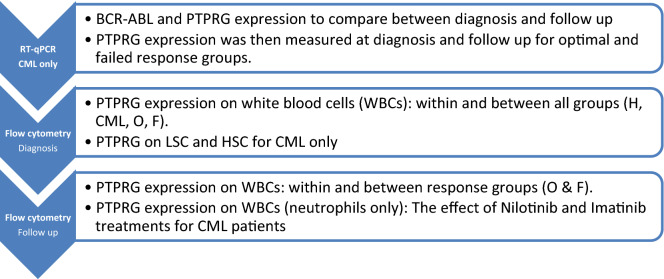
Presentation of results.

Figure 1 shows the sequence of results and analysis that were performed using two techniques at different time points (diagnosis and follow up) for different comparisons.

### Expression of *BCR-ABL1* and *PTPRG* mRNA in whole white blood cells using RT-qPCR

**Figure 2:**
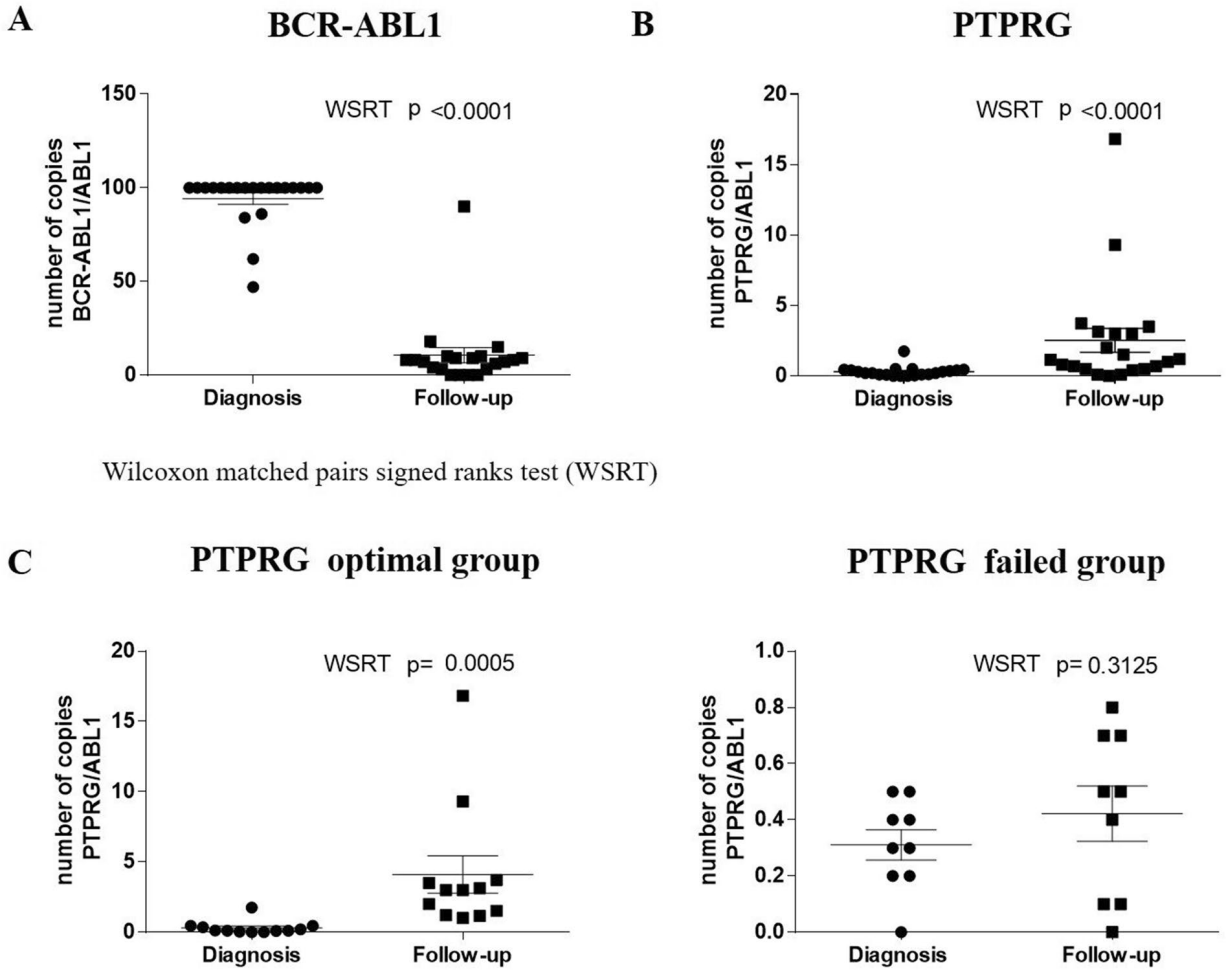
mRNA levels of *BCR-ABL1* and *PTPRG* in CML patients at diagnosis and follow up. (**a**) BCR-ABL1 transcript levels at diagnosis and follow up (mean of post-test ranks = 10 and mean of pre-test ranks = 94.24, Z = − 4.018, *p *˂ 0.001). (**b**) PTPRG transcript levels at diagnosis and follow up (mean of post-test ranks = 2.53 and mean of pre-test ranks = 0.3, Z = − 3.50, *p *˂ 0.001) (**c**) mRNA transcripts level of PTPRG in the optimal response and failed treatment groups. The mRNA level of PTPRG in the optimal response group was significantly higher when compared with diagnosis, while this significance was lost in the CML group. The Y-axis represents number of PTPRG/ABL1 of mRNA copies, while the X-axis represented timelines at diagnosis and mean of follow up. P values were derived from the WSRT test.

The expression levels of *BCR-ABL1* and *PTPRG* mRNA levels in CML patients at diagnosis and follow up were determined by RT-qPCR and the results are presented in Fig. 2. As the results of Cohn’s d coefficient analysis show, there was a “huge” and “large” effect size on *BCR-ABL1* (Cohen's d = 5.05) and *PTPRG* transcripts (Cohen's d = 0.81) following treatment with TKIs. A significant difference was found in the mean levels of *BCR-ABL1* mRNA at diagnosis and at follow up (WSRT *p *˂ 0.001, Fig. 2a) and *PTPRG* mRNA at diagnosis and at follow up (WSRT *p *˂ 0.001, Fig. 2b). In contrast to *BCR-ABL1* mRNA, which had significantly higher levels at diagnosis compared to follow up, PTPRG mRNA expression levels were found to be significantly lower at diagnosis compared to follow up. There was also a moderate negative correlation between *BCR-ABL1* at diagnosis and *PTPRG* at follow up (*r*_*s*_ (21) = − 0.422, p = 0.028).

Moreover, the *PTPRG* transcript was also assessed to compare between optimal and failed CML groups. The results showed that the optimal response group had significantly higher *PTPRG* expression during follow up (WSRT p ˂ 0.0005) compared to diagnosis time-point, while no significant difference was found amongst the failed group at diagnosis and during follow up (WSRT p = 0.312, Fig. 2c).

### PTPRG expression levels on white blood sub-populations (WBCs) of Healthy individuals and CML patients at diagnosis using flow cytometry

**Figure 3:**
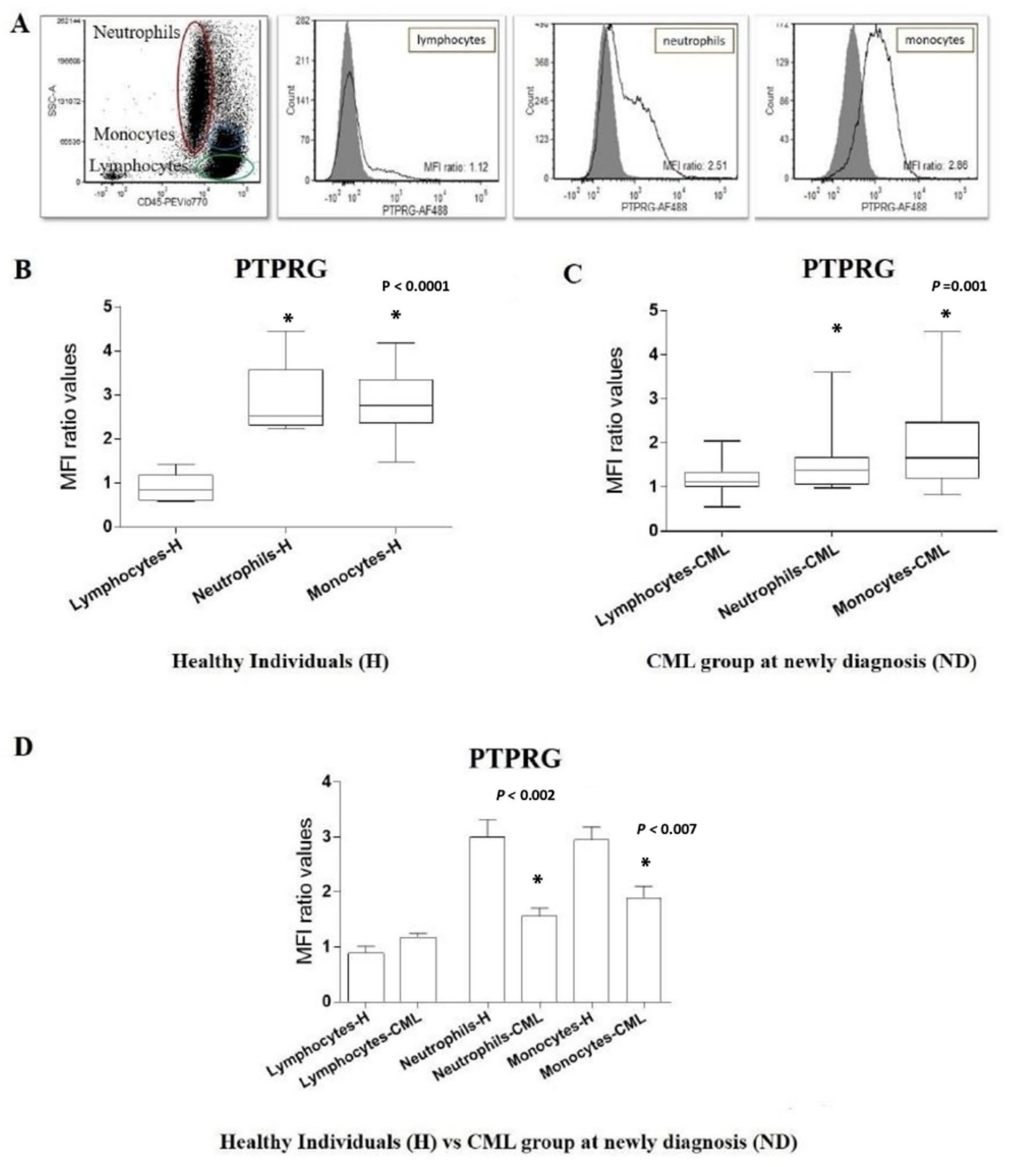
PTPRG protein expressions on sub-population of white blood cells of healthy individuals (H) and CML patients (ND)*.* (**a**) Level of PTPRG expression on sub-population of white blood cells of (H). (Y-axis) refers to count numbers of peripheral blood cells recorded in each sub-population at flow cytometry acquisition. The expression of PTPRG was reported in Mean Fluorescent Intensity (MFI). (**b**) The mean of PTPRG expression (H) on both neutrophils (mean = 3.0), monocytes (mean = 2.8) was significantly higher when compared with lymphocytes (mean = 0.89). (**c**) The median of PTPRG expression (ND) on monocytes (median = 1.7); neutrophils (median = 1.5) was significantly higher when compared with lymphocytes (median = 1.14). (**d**) PTPRG expression on neutrophils and monocytes was significantly lower in CML (ND) patients in comparison with healthy individuals (H).

**Figure 4:**
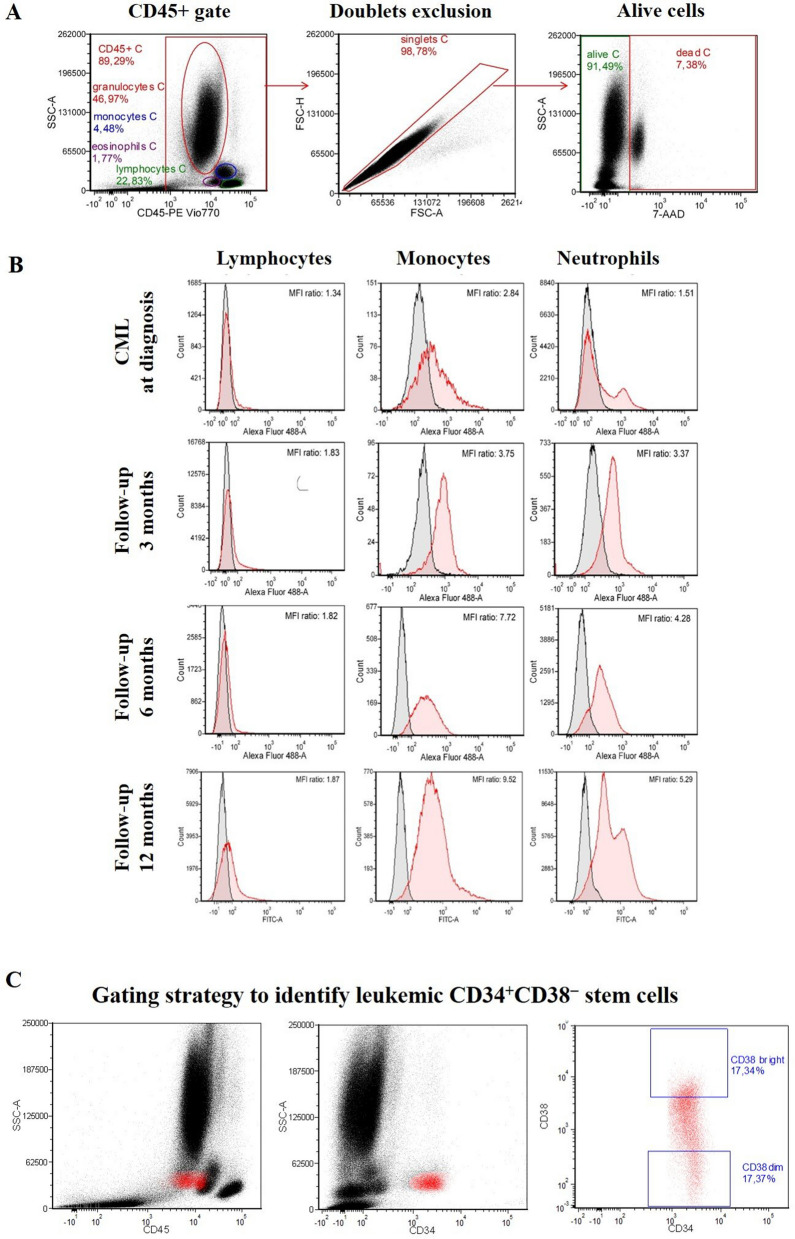
Gating strategies of flow cytometric analysis of PTPRG protein and its expression during the treatment plan. (**a**) Gating strategies of PTPRG expression on the sub-population of white blood cells. Neutrophils (red color) have an intermediate level of CD45 and high side-scattered light (SSC); Monocytes (blue color) have a slightly higher level of CD45 expression and intermediate SSC while lymphocytes (green color) have the highest level of expression of CD45 but the lowest level of SSC. Doublets discrimination and exclusion of dead cells using 7-AAD staining (red rectangle) allow an easier identification of positively stained populations. In comparison the lower population was the target of part of the leukemic stem cell. (**b**) Neutrophils and Monocytes showed a low level of PTPRG expression at the time of diagnosis. PTPRG restored its expression, at least in part of the sub-population of white blood cells followed by TKIs therapy. Of note, lymphocytes remained at a low level acting as an internal control. Follow up time points F1, F2 and F3 were 3, 6, and 12 months of successful TKIs as per ELN timelines. The MFI values were obtained by calculating the ratio differences between the signals derived from the signal of mAB TPγ B9-2 and irrelevant mouse IgG1. (**c**) Gating strategy to identify leukemic CD34+CD38− stem cells. For myeloid progenitors and its sub-population, we targeted 15–20% upper and lower population of CD34 (red color) with CD38, with the upper population corresponding to the target of interest (hematopoietic stem cells). In comparison the lower population correspond to part of the leukemic stem cell.

Next, the expression levels of PTPRG protein were determined in the WBCs sub-populations of healthy and CML patients using flow cytometry and the results are presented in Figs. 3 and 4. A significant difference was found in the expression levels of PTPRG between different WBC sub-population (neutrophils, monocytes, and lymphocytes) [F (3, 5) = 19.15, *p *˂ 0.0001]. PTPRG protein expression levels were found to be higher on neutrophils and monocytes when compared to lymphocytes in both healthy individuals (Fig. 3a,b) and CML patients at diagnosis (Fig. 3c).

When comparing healthy individuals against CML patients, PTPRG protein expression levels were significantly higher on neutrophils (*U* = 30, *p* < 0.002) and monocytes (*U* = 24, *p* < 0.007) amongst healthy individuals in comparison to CML patients at diagnosis (Fig. 3d). A significant difference was also found in the expression level of PTPRG on neutrophils between the healthy, optimal and failed groups (H = 14.94, df = 3, *p* = 0.001). The optimal response group had higher expression PTPRG in their neutrophils compared to the healthy group (*U* = 3, *p* = 0.004), and the failed groups had higher expression of PTPRG than the healthy group (*U* = 7, *p* = 0.004).

### Expression levels of PTPRG protein on neutrophils and monocytes in optimal and failed groups at follow up determined by flow cytometry

**Figure 5:**
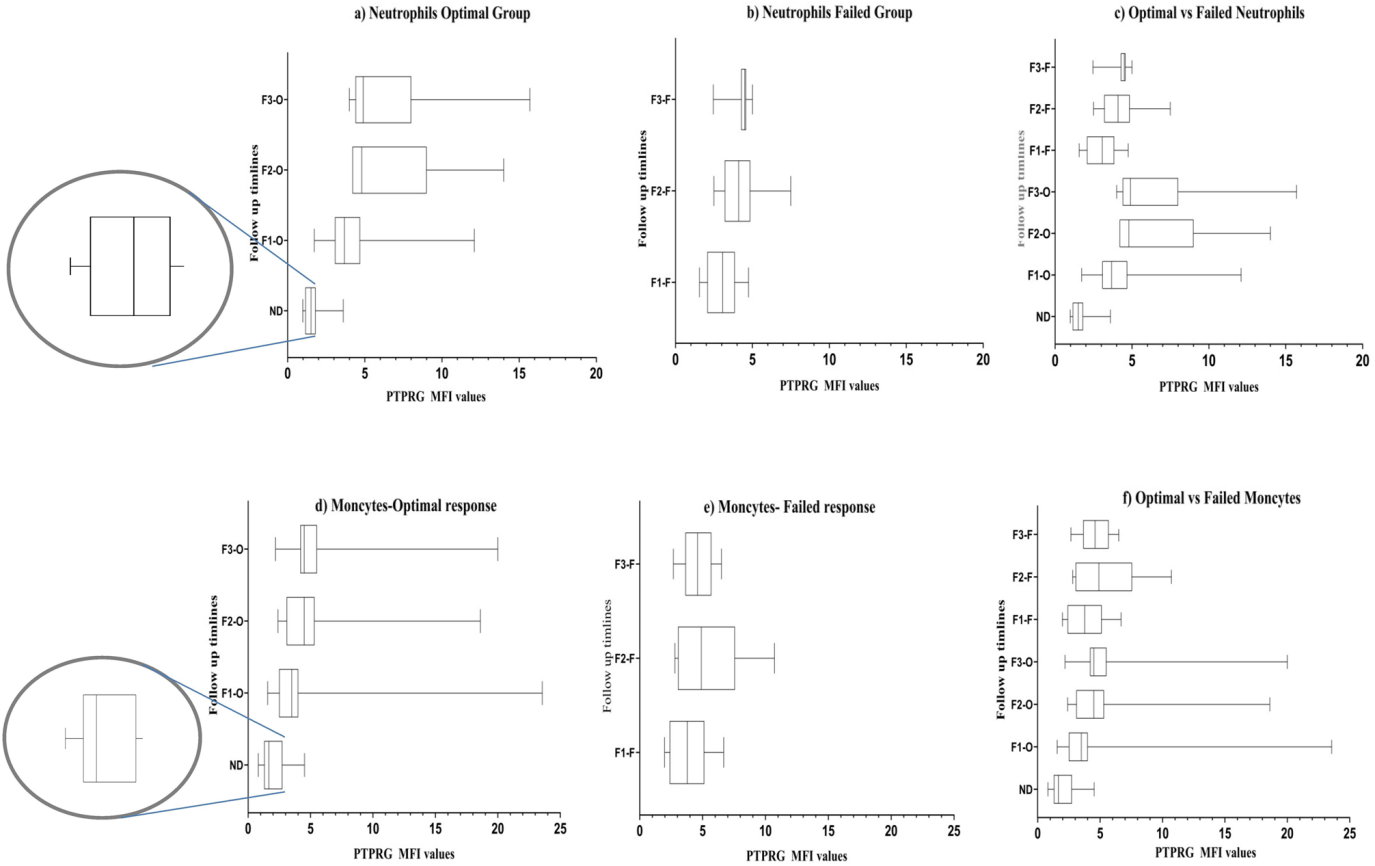
PTPRG protein expressions upon stratification of CML patients’ response in white blood cells and its sub-population. PTPRG level on neutrophils at diagnosis showed a median of 1.50 with negative skewness. (**a**) The restoration level of PTPRG on neutrophils of the optimal response group was greater in follow up periods compared to diagnosis phase, median Follow-up 1 = 3.68; median Follow-up 2 = 4.8; median of Follow-up 3 = 4.9. Histogram showed positive skewness for later follow up periods. (**b**) PTPRG expression on neutrophils of failed response increased significantly by the first follow up compared to the time of diagnosis; median of Follow-up 1 = 3.065; median of Follow-up 2 = 4.0; median of Follow-up 3 = 4.5. Histogram of 1st and 2nd follow-ups showed a normal distribution (Shapiro–Wilk test, W), while the 3rd follow up showed negative skewness. (**c**) Hodges–Lehmann estimator between optimally and failed groups on neutrophils; 1st follow up: − 0.78, 2nd follow up: − 1.35 and 3rd follow up: − 0.9. PTPRG level on monocytes at diagnosis showed a median of 1.7 with positive skewness. (**d**) The restoration of PTPRG level on monocytes of optimal response was greater in follow up periods when compared to diagnosis phase, medianFollow-up 1 = 3.5; median Follow-up 2 = 4.5; median Follow-up 3 = 4.5. Histogram of 1st and 2nd follow-up periods showed negative skewness, while the 3rd follow-up showed positive skewness. (**e**) The restoration of PTPRG level on monocytes in the failed response group showed a similar scenario as the optimal response group with a median Follow-up 1 = 3.8; median Follow-up 2 = 4.9; median Follow-up 3 = 4.6. The histogram of follow up periods showed a normal distribution (Shapiro–Wilk test, W). (**f**) Hodges–Lehmann estimator between optimally and failed groups on monocytes 1st follow up: − 0.34, 2nd follow up: − 0.35 and 3rd follow up: − 0.35. *Referred p-value was statistically significant. *O* optimal response, *F* failed group, *ND* new diagnosis, *F1* 1st follow up, *F2* 2nd follow up, *F3* 3rd follow up.

The PTPRG expression level on WBCs was also re-assessed during the follow up for both optimal and failed CML groups. There was a significant difference between the expression of *PTPRG* on the neutrophils (*χ*^2^ (2, 11) = 13.82, *p* = 0.001) and monocytes (*χ*^2^ (2, 11) = 10.09, *p* = 0.006) during the follow-up time points in the optimal response group. For the neutrophils, there were significant differences between 1st follow up (median: 3.68) and 2nd follow up (median: 4.8, Z = − 2.93, *p *˂ 0.001) and 1st follow up and 3rd follow up (median: 4.9) (Z = − 2.67, *p *˂ 0.001), but no significant differences between the 2nd and 3rd follow up (Z = − 0.89, *p* = 0.37, Fig. 5a). However, for the monocytes there were only significant differences in its expression levels between 1st follow up (median: 3.5) and 3rd follow up (median: 4.5, Z = − 2.05, *p* = 0.004, Fig. 5). There were no significant differences between the follow up time-points in relation to the expression of PTPRG on the lymphocytes in the optimal response group (*χ*^2^ (2, 11) = 2, *p* = 0.6).

In the failed response group, there were significant differences in the PTPRG expression levels between 1st follow up (median: 3.1) and 2nd follow up (median: 4.0) (Z = − 2.8, *p *˂ 0.005) and 1st and 3rd follow up (median: 4.5, Z = − 2.6, *p* = 0.009), but no significant difference between 2nd and 3rd follow up (Z = − 1.33, *p* = 0.19, Fig. 5b). For the monocytes, there were significant differences between only the 1st follow up (median: 3.8) and 3rd follow up (median: 4.6), (Z = − 2.7, *p* = 0.027, Fig. 5). In contrast, there were no significant differences in the expression levels of PTPRG protein between the follow up time-points in the failed response group (*χ*^2^ (2, 10) = 2, *p* = 0.8).

When PTPRG expression was compared on neutrophils of optimal and failed group, there was no significant difference in the 1st follow up (*U* = 39, *p* = 0.26). On the other hand, there were significant differences between the two response groups at the 2nd follow up (*U* = 25.5, *p* = 0.036) and the 3rd follow up (*U* = 28.0, *p* = 0.05) (Fig. 5c). No significant differences in the monocytes of the optimal and failed groups were found between the follow up time points (1st follow up (*U* = 46.0, *p* = 0.53), 2nd follow up (*U* = 49.5, *p* = 0.7) and 3rd follow up (*U* = 48.0, *p* = 0.62)) (Fig. 5f).

In CML patients, the impact of treatment (Nilotinib vs. Imatinib) on neutrophils’ PTPRG expression significantly differed over time (Nilotinib: *χ*^2^ (3, 8) = 18.45, *p* = 0.001; Imatinib: (*χ*^2^ (3, 12) = 32.9, *p* = 0.001). There was a significant treatment effect between diagnosis and 1st follow up (only for Nilotinib treatment) (*U* = 22, *p* = 0.047), between diagnosis and 2nd follow up (both treatments), and between diagnosis and 3rd follow up (both treatments). On the other hand, only Imatinib treatment was significantly more effective at the 3rd follow up compared to the 1st follow up (Table 2B).

### *PTPRG* expression on myeloid progenitors within the optimal and failed groups

After analysis of mature population (WBCs), we studied PTPRG expression in two subpopulations of progenitors’ cells, leukemic stem cell (LSCs) and hematopoietic stem cells (HSCs). The results show that there was no significant difference in PTPRG expression at the time of diagnosis (*U* = 31, *p* = 0.183), although the mean of PTPRG expression level on (LSC) (medium Glass' Delta effect size: 0.52) was higher than HSC (small Glass’ Delta effect size: 0.17) (Table 3). Furthermore, the mean rank of PTPRG expression on LSC at the time of diagnosis in the failed group was significantly higher than the optimal group (*U* = 12, *p* < 0.004).

**Table 3:**
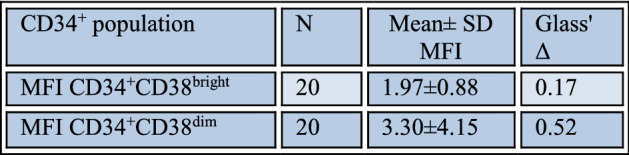
The expression level of PTPRG in the sub-population of myeloid progenitor cells.

## Discussion

In this study, for the first time we investigated the changes in the PTPRG protein level in CML patients at the State of Qatar determined by flow cytometry using a unique monoclonal antibody against the external domain of PRPRG. We confirmed that restoration levels of *PTPRG* were significantly greater during the follow-up period following treatment with BCR-ABL1 TKIs compared to the time of diagnosis (Fig. 4b). Moreover, stratification of CML patients’ response into optimal and failed treatment groups showed that the expression level of *PTPRG* to be significantly higher in the optimal response group when compared to the failed group, utilizing both q-PCR and flow cytometry techniques. Our results also confirmed our earlier findings using RQ PCR, in which 33 CML patients had been studied and the mRNA level of PTPRG was found to be significantly high in patient achieving Major Molecular Response (MMR), while this was not the case in non-responsive cases^17^.

There is currently no clear associations between the expression of a biomarker at mRNA level and its translation at protein level and the link between the two parameters ranged from 40% up to 90%^18–20^. On the contrary, other publications reported a low correlation between levels of mRNA and protein expression due to many factors including steady state, degradation, proteomics, and transcriptomics factors^21–23^. In the current study, we investigated the expression levels of PTPRG at both the mRNA and protein levels in parallel. While we did not find any significant differences in the expression of PTPRG at the mRNA level in the failed group (WSRT *p* = 0.312) (Fig. 2c), there were significant differences in the PTPRG protein expression level on neutrophils population in CML of the optimally treated group (Fig. 5c). Our results showed the importance of studying the expression levels of various biomarkers at both mRNA and protein levels.

In this study, we found that the restoration of the *PTPRG* level to be significantly higher on the neutrophils population in the optimal response group, when compared to the failed group at two follow up points (2nd and 3rd follow up) (Fig. 5c). On the other hand, there was not a significant difference in the expression of *PTPRG* on the monocytes population between the optimal and the failed groups at any follow up point (Fig. 5f). Our data also showed that the restoration of PTPRG expression on neutrophils was drug-dependent as the expression of PTPRG was restored earlier with Nilotinib when compared to Imatinib Mesylate. This observation might reflect the superior potency of Nilotinib as BCR-ABL1 TKI, which was reported to be 20–50 times more potent than Imatinib, and its ability to achieve complete superior response compared to Imatinib Mesylate^24^.

In another study, Naoto and his colleagues developed a fluorescence in situ hybridization (FISH) technique named “Neutrophil-FISH” that has the ability to classify CML cohort to responder and non-responder to Imatinib Mesylate^25^. In the current study, we reported the ability of mAB TPγ B9-2 to record changes in expression of PTPRG on neutrophils by flow cytometry technique and consequently classify the CML patients in the same manner.

The restoration of PTPRG expression reached the level recorded in healthy individuals and this may be explained by the recovery of healthy hematopoiesis in these subjects due to that TKIs having a large impact on the *PTPRG* gene (Cohen’s d = 0.81). On the other hand, there was still a significant difference between levels of PTPRG in healthy, optimally, and failed groups. This may be explained by overexpression of PTPRG to overcome the uncontrolled BCR-ABL1 kinase activity.

In relation to expression of PTPRG on myeloid lineage, our previous study have shown a low level of PTPRG expression in CML on myeloid lineage at the time of diagnosis^17^. Interestingly, in this study, we found the mean PTPRG expression level to be higher in hematopoietic stem cells when compared to leukemic stem cells. The Glass' Delta effect size equation showed a small effect size of level of PTPRG at HSCs, as compared to a medium effect size of the level of PTPRG on LSCs (Table 3). Additionally, the expression of PTPRG on LSCs was significantly higher in the failed group when compared to the optimal response. The result was matched with the fact that leukemic stem cells have a unique cell surface profile, which is different from that of hematopoietic cells^26^. Furthermore, LSCs had the ability to self-renewal^27^, and LSCs had a signatory high expression of a gene, which is independently associated with adverse outcomes of treatment^28^ and could predict the prognosis of the disease^29^. A recent study documented that *BCR-ABL1* transcripts maybe not transcribed by LSC of CML patients^30^.

Finally, primary resistance and acquired resistance to treatment with BCR-ABL1 TKIs can occur in some patients with CML^31–34^. In Qatar, a significantly higher percentage of CML patients develop resistance to TKIs compared to the other parts of the world^12,35^. This may be due to the fact that most studies in CML management were focused on one component (tyrosine kinases), while the other arm (tyrosine phosphatases) has not yet received equivalent attention.

In summary, in this study, we have shown that the expression of PTPRG, a tumor suppressor gene, is suppressed in CML patients and this can be restored following treatment with TKIs to levels observed in healthy controls. We have also shown that restoration levels were greatest in optimal responders and occurred earlier with nilotinib compared with imatinib. Taken together, our results support that determination of PTPRG expression level by flow cytometry as a new biomarker of response to treatment with BCR-ABL1 TKIs is a useful tool for studying its role in tumor progression and predicting the response to therapeutic interventions and clinical management in patients with CML.

## Materials and methods

### Patient recruitment, characteristics and sample collection

#### Participants

A total of 21 adult CML patients whom were regularly followed up and treated with Tyrosine Kinase Inhibitors (Imatinib and Nilotinib) in the National Center for Cancer Care & Research clinics were recruited into this study. Peripheral blood samples were collected in EDTA tubes, where 63 subsequent samples were collected according to the ELN treatment timepoints. Seven matched healthy individuals (H) with normal complete blood count (CBC) and who were negative for *BCR-ABL1* translocation were included in this study.

#### Patients’ blood samples, procedure, and ethics

Informed consent was obtained from all participants. The study was approved by both the Ministry of Public Health (MOPH) and the Institutional Review Board (IRB) of Hamad Medical Corporation (HMC) (Qatar) (Project No. 11118/11) and Kingston University London’s ethical committee (UK). This study adhered to the World Medical Association’s Declaration of Helsinki (1964–2008) for ethical human, research including confidentiality, privacy, and data management.

Blood samples were collected from newly diagnosed patients (ND) at day zero and before starting any tyrosine kinase inhibitor (TKI) or Hydroxyurea treatment. For failed/relapsed patients’ (F), blood samples were collected at the time of failure. The CML patients’ response to treatment was assessed based on the hematologic, cytogenetic, and molecular responses according to the European LeukemiaNet (ELN; 2013). The absence of one of the following was considered as treatment failure: abnormal complete blood count and/or Ph^+^ > 95% by 3 months, BCR-ABL1 > 10% and/or Ph^+^ > 35% by 6 months, or BCR-ABL1 > 1% and/or Ph^+^ > 0 by 12 months treatment^36^. The Optimal response group (R) was defined as achieving hematological, molecular, and cytogenetic remission within the time points of ELN guidelines.

#### Determination of *PTPRG* and *BCR-ABL1* transcripts by RT-qPCR

A RT-qPCR was carried out to measure the mean levels of mRNA of *PTPRG* and *BCR-ABL1* at 3, 6 and 12 months. Plasmids carrying PTPRG full length were utilized to standardize the number of copies measured by PCR as previously described^9,17,35,37,38^. IPSOGEN *BCR-ABL1* Mbcr IS-MMR Kits CE kit (Cat No. 670823) was utilized to quantify *BCR-ABL1* according to the international scale (IS). RT-qPCR and Flow cytometry measurements were done simultaneously.

#### Flow cytometry

Isolated white blood cells (2 × 10^6^) were diluted into 200 μl phosphate-buffered saline (PBS1×) and transferred to FACs tubes (two tubes for isotope and PTPRG expression). The FACs tubes were placed on ice, and 5 μl CD45 primary antibody (Becton Dickinson international company (BD)-PE-CY7 anti-Human mouse Catalog No. 557748), 5 μl of CD34 antibody (BD-BV421 mouse anti-Human Catalog No. 562577), and 20 μl CD38 antibody (BD-APC labeled anti-Human Catalog No. 555462) were added and incubated in the dark for 20 min at 4 °C according to supplier’s recommendations. After washing, 20 μl of mouse IgG1-AF 488 antibody (BD-mouse IgG1-AF 488 (20 μg) Catalog No. 557782) and 1 μl of TPγ B9-2-AF488 antibody (1 μg) were added and incubated in the dark for 40 min then washed twice with cold PBS at 200 × *g* for 5 min at 4 °C. The samples were then analyzed by flow cytometry (BD LSR FORTESSA cell analyzer), where 500,000–1,000,000 numbers of events were targeted at acquisition records.

Flow cytometry was performed on peripheral blood cells (PBCs) to characterize the expression of PTPRG on lymphocytes, monocytes, and neutrophils (LMN). The gating strategy of the flow cytometry experiment aimed at monitoring PTPRG expression in the major populations represented by neutrophils, monocytes and lymphocytes at diagnosis and during the follow up phase of TKIs treatment (Fig. 4a,b).

L,M,N were gated with CD45 and side-scattered light (SSC). Neutrophils were characterized by intermediate CD45 and high SSC. Monocytes have a slightly higher CD45 expression and intermediate SSC, while lymphocytes have the highest expression of CD45 and the lowest level of SSC. Additionally, we targeted the level of *PTPRG* on myeloid lineages via the CD34 antibody and their sub-populations (CD38^+/−^). CD34^+^/CD38^−^ (Dim) targeted leukemic stem cells (LSCs), while CD34^+^/CD38^+^ (bright) targeted hematopoietic stem cells (HSCs). The expression of PTPRG was reported in Mean Fluorescent Intensity (MFI) that represents the difference in expression between Target of Interest (TOI) and an isotype control.

### Statistical analysis

All statistical tests were performed using SPSS v26. Changes in mRNA levels of (*BCR-ABL1* & *PTPRG*) over timeline were analyzed with the Wilcoxon matched pairs signed ranks test (WSRT). The effect size of TKIs was reported and assessed on both genes (*BCR-ABL1* & *PTPRG*) via the Cohen’s d coefficient for which d = 0.2 was considered a ‘small’ effect size, 0.5 a 'medium' effect size, 0.8 a 'large' effect size and 2.0 a “huge” effect size^39,40^. Additionally, a Spearman’s rank-order correlation was run to determine the relationship between the two genes.

The distribution of the data was tested for normality with the Shapiro–Wilk test (W), and Skewness and normally distributed data was tested by the One-way Analysis Of Variance (ANOVA) and Dunnett’s multiple comparison tests. Non-normally distributed data was tested by Friedman and Dunnett’s multiple comparison tests. On the other hand, the Mann Whitney test was performed to analyze the difference between normally and non-normally distributed data. Changes in expression of PTPRG in CML patients over timeline were analyzed with Friedman and Wilcoxon signed ranks (WSRT) tests and a pairwise comparison with Hodges–Lehmann estimator. Finally, Kruskal–Wallis, along with Mann Whitney tests, were employed to examine the changes of PTPRG among healthy (H), optimally (R), and failed (F) groups.

### Consent for publication

Consent for publication was obtained through ethics approval and consent to participate.

## Data Availability

This is a research article, and all data generated or analyzed during this study are included in this published article. Please contact the corresponding author for data and material requests. *Monoclonal Antibody for Diagnosing Chronic Myeloid Leukemia Patent* Hamad Medical Corporation sponsored the patent process of the antibody and assignee: Qatar foundation. Provisional US patent application number: US62373322.

## Abbreviations

AF: Alexa fluro
AP: Accelerated phase
APC: Allophycocyanin
ANOVA: Analysis of variance
BC: Blast crisis phase
BD: Becton and Dickinson biosciences company
PE-CY7: A tandem fluorochrome consisting of R-phyco erythrin
BV: Brilliant violet
CBC: Complete blood count
CML: Chronic myeloid leukemia
CP: Chronic phase
EDTA: Ethylenediaminetetraacetic acid
ELN: European LeukemiaNet
F: Failed treatment
FACs: Fluorescence-activated cell sorting
FDA: Food and Drug Administration
FISH: Fluorescence in situ hybridization
FU: Follow up
H: Healthy individuals
HMC: Hamad Medical Corporation
HSCs: Hematopoietic stem cells
IM: Imatinib Mesylate
IgG: Immunoglobulin G
IS: International scale
LSCs: Leukemic stem cells
L: Lymphocytes
L,M,N: Lymphocytes, monocytes, and neutrophils
M: Monocytes
mAb: Monoclonal antibody
MFI: Mean fluorescent intensity
MMR: Major molecular response
MOPH: Ministry of Public Health
mRNA: Messenger RNA
N: Neutrophils
ND: New diagnosis
PCR: Polymerase chain reaction
Ph+: Philadelphia chromosome
PBCs: Peripheral blood cells
PTPRG: Protein tyrosine phosphatase receptor gamma
PBS: Phosphate-buffered saline
RT-qPCR: Reverse transcription PCR
SSC: Side scatter
SD: Standard deviation
SPSS: Statistical Package for the Social Sciences
W: Shapiro–Wilk
TKIs: Tyrosine kinase inhibitors
WSRT: Wilcoxon Signed-Ranks Test
